# Prevalence and Mortality-Related Factors of Multiple Myeloma in Taiwan

**DOI:** 10.1371/journal.pone.0167227

**Published:** 2016-12-01

**Authors:** Jia-Hong Chen, Chi-Hsiang Chung, Yung-Chih Wang, Shun-Neng Hsu, Wen-Yen Huang, Wu-Chien Chien

**Affiliations:** 1 Division of Hematology/Oncology, Department of Medicine, Tri-Service General Hospital, National Defense Medical Center, Taipei, Taiwan; 2 Graduate Institute of Clinical Medicine, College of Medicine, Taipei Medical University, Taipei, Taiwan; 3 School of Public Health, National Defense Medical Center, Taipei, Taiwan; 4 Taiwanese Injury Prevention & Safety Promotion Association, National Defense Medical Center, Taipei, Taiwan; 5 Division of Infectious Diseases and Tropical Medicine, Department of Internal Medicine, Tri-Service General Hospital, National Defense Medical Center, Taipei, Taiwan; 6 Institute of Clinical Medicine, National Yang-Ming University, Taipei, Taiwan; 7 Division of Nephrology, Department of Medicine, Tri-Service General Hospital, National Defense Medical Center, Taipei, Taiwan; 8 Department of Radiation Oncology, Tri-Service General Hospital, National Defense Medical Center, Taipei, Taiwan; 9 National Defense Medical Center, Tri-Service General Hospital, Department of Medical Research, Neihu District, Taipei City, Taiwan; 10 National Defense Medical Center, School of Public Health, Neihu District, Taipei City, Taiwan; Kaohsiung Medical University Hospital, TAIWAN

## Abstract

In this retrospective cohort study based in Taiwan, we reported the current epidemiology of patients with multiple myeloma and analyzed the factors affecting mortality. We identified 7285 patients with newly diagnosed multiple myeloma (MM) between 1997 and 2013 in Taiwan. Privileges data from the National Health Institute Research Database was used, as it is made readily available to the public in electronic format for research purposes. From 1997 to 2013, the average incidence of MM per 100,000 people was 1.83. The mortality accounted for an average of 0.44 per 100,000 deaths. In all 7285 inpatients with MM, the proportion of male patients was greater than that of female (59.90% vs. 40.10%); the mean age was 68.71 years with the proportion of those >55 years of age was 85.11%; and the proportion of a catastrophic illness was 66.51%. The death risk of the inpatient dialysis group was 3.044 times that of patients without dialysis (P <0.001). Moreover, the risk of death to men in the hospital setting was 1.162 times that of women (P = 0.012), and in the group of patients aged >55 years, the risk of in-hospital death was 1.511 times more than that in those aged ≤55 years (P <0.001). The risk of hospital death due to catastrophic illness was 1.347 times that of a non-catastrophic illness (P <0.001). Male patients and those >55 years of age had the most common prevalence of MM in Taiwan. Hemodialysis treatment, male sex, old age, and catastrophic illness were independent predictors of hospital mortality in patients with MM.

## Introduction

Multiple myeloma (MM) is a cancer of plasma cells, white blood cells that are naturally responsible for producing antibodies [1–2]. Worldwide, MM resulted in about 74,000 deaths in 2010, up from 49,000 in 1990 [3]. These numbers are established on assumptions made using data from 2011, which estimated the prevalence at 83,367 people, the incidence at 6.1 per 100,000 people per year, and the mortality at 3.4 per 100,000 people per year [4]. Asians have the lowest reported incidence of MM, with men affected slightly more than women do.

The reported age-adjusted incidence of MM per 100,000 people around the world is 0.5 in Hawaiian Japanese men [5–6]. However, recent reports have suggested that the incidence of MM is increasing in some Asian countries [7–8].

The Taiwan National Health Insurance (NHI) system is launched in 1995, currently covers 99% of the population of 23 million people [6]. In 1998, nearly the NHI protected 99% of the Taiwanese. From 1997 to 2013, NHI program inpatients accounted for more than 15 million people. This nationwide database from Taiwan provides an opportunity to evaluate the epidemiology and survival outcomes of numerous MM patients.

The cohort study by Huang [8] is the first report to describe the epidemiology of MM in Chinese populations comprehensively. However, the study was done 10 years ago based on the database of Taiwan National Cancer Registry. The aims of this study were to present expressive epidemiology of MM in Taiwan, a country populated by 23 million Chinese located in southeastern Asia, and to provide main epidemiological data in this population between 1997 and 2013. The potential effects of patient age, gender, low-income household, catastrophic illness [9], admission season, outpatient location, urbanization level, hematology and oncology departments, surgery, length of days, and medical cost (NT$) on the change in incidence of MM in Taiwan were examined.

## Materials and Methods

### Data Sources and Study Population

The National Health Research Institute (NHRI) creates all privileges data from the National Health Institute Research Database (NHIRD) available to the public in electronic format for research purposes [10]. We recycled two data files: NHIRD, and all inpatient records for cancer care. We applied the codes of the International Classification of Diseases, 9th Revision, Clinical Modification (ICD-9-CM) to recover diagnosis information.

### Ethical Considerations

The NHIRD encrypts personal patient information to keep privacy and provides researchers with anonymous identification numbers associated with relevant claim information, including patients’ sex, dates of birth, medical services utilized, and prescriptions. Patient consent is not required for accessing the NHIRD. The Institutional Review Board of TSGH approved this study. Our IRB specifically waived the consent requirement.

### Study Participants

Patients who were disclosed for the Catastrophic Illness Patient Database (CIPD) required insurance approval, including inpatient cases. We recognized 7285 patients newly diagnosed with MM (ICD-9 code 203.0) from the CIPD from 1997 to 2013 as the MM cohort. The date of MM diagnosis was established as the index date for beginning the measurement of follow-up person-years. All patients were followed up until death, censored for loss of follow-up, withdrawal from the insurance system, or until the end of 2013. The confirmation of death events was based on CIPD and inpatient records in the NHIRD.

### Statistical Analysis

Distributions of definite sociodemographic factors, including age (≤55, >55 years), residential geographic area (Northern, Central, Southern, eastern Taiwan, and Outlets islands), inpatient season, catastrophic illness (with, without), and low-income household (with, without) were displayed between male and female MM patients. We calculated hazard ratios (HRs) and the 95% confidence interval (CI) using the Cox proportional hazards model to assess the HR of mortality in MM patients. The multivariate Cox proportional hazards model was performed to measure the mortality-association risk factor in MM patients after adjustment for MM treatment and sociodemographic characteristics. SAS version 9.1 (SAS Institute, Cary, NC, USA) was used for data analyses; P < .05 was reflected statistically significant.

## Results

**Fig 1:**
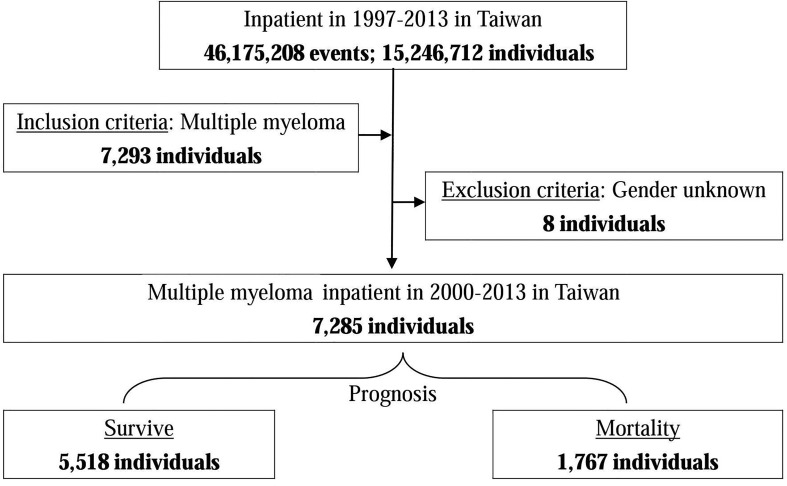
Flowchart of the study sample selection from the National Health Insurance Research Database in Taiwan. Multiple myeloma: ICD-9-CM 203.0; Inclusion criteria: Multiple myeloma; Exclusion criteria: Gender unknown.

Overall, from 1997 to 2013 in Taiwan, 7285 patients were hospitalized with MM mean their all admissions after MM was diagnosed, of which 5518 people (75.74%) survived after hospitalization, but of which 1767 people (24.26%) died post-treatment following a hospital stay (Fig 1). From 1997 to 2013, the average incidence per 100,000 people was 1.83. The mortality accounted for an average of 0.44 per 100,000 deaths.

In 7285 in-patients with MM, the proportion of male patients was greater than that of female (59.90% vs. 40.10%); mean age was 68.71 years and the proportion of patients >55 years of age was 85.11%. The proportion of low-income households was 0.74%, but there are not different form survive group and mortality group (P = 0.561). The proportion of a catastrophic illness, a severe illness requiring prolonged hospitalization or recovery [9], was 66.51%; the Charlson comorbidity index (CCI) with disease index score was 0.65 points, but there are not different form survive group and mortality group (P = 0.495); and there was a higher proportion of autumnal hospitalization (28.00%). There were a higher proportion of those living in the north (49.57%) and in the city (47.15%). A higher proportion of patients were hospitalized for medical treatment at the Medical Center compared with specifically at the Department of Hematology and Oncology (63.21% and 44.94%, respectively). Those with surgery at their disposal accounted for 18.42%, the average length of hospitalization was 56.79 days, and medical expenses were 387,795 yuan on average (Table 1).

**Table 1:** Characteristics of multiple myeloma inpatients.

Prognosis	Total		Survive		Mortality		P-value
Variables	n	%	n	%	n	%	
Total	7,285		5,518	75.74	1,767	24.26	
Hemodialysis							<0.001***
Without	2,814	38.63	2,486	45.05	328	18.56	
With	4,471	61.37	3,032	54.95	1,439	81.44	
Gender							0.003**
Male	4,364	59.90	3,256	59.01	1,108	62.71	
Female	2,921	40.10	2,262	40.99	659	37.29	
Age (years)	68.71±12.38		68.28±12.56		70.02±11.73		<0.001***
Age group (years)							<0.001***
≦55	1,085	14.89	866	15.69	219	12.39	
>55	6,200	85.11	4,652	84.31	1,548	87.61	
Low-income household							0.561
Without	7,231	99.26	1,754	99.26	5,477	99.26	
With	54	0.74	13	0.74	41	0.74	
Catastrophic illness^#^							<0.001***
Without	2,440	33.49	2,010	36.43	430	24.34	
With	4,845	66.51	3,508	63.57	1,337	75.66	
CCI_R	0.65±0.98		0.65±0.98		0.67±0.98		0.495
Inpatient season							<0.001***
Spring (March-May)	1,606	22.05	1,164	21.09	442	25.01	
Summer (June-August)	1,854	25.45	1,382	25.05	472	26.71	
Autumn (September-November)	2,040	28.00	1,606	29.10	434	24.56	
Winter (December-Februrary)	1,785	24.50	1,366	24.76	419	23.71	
Outpatient location							<0.001***
Northern Taiwan	3,611	49.57	2,575	46.67	1,036	58.63	
Middle Taiwan	1,788	24.54	1,469	26.62	319	18.05	
Southern Taiwan	1,541	21.15	1,200	21.75	341	19.30	
Eastern Taiwan	324	4.45	255	4.62	69	3.90	
Outlets islands	21	0.29	19	0.34	2	0.11	
Urbanization level							<0.001***
High	2,927	40.18	2,213	40.11	714	40.41	
Middle	3,435	47.15	2,542	46.07	893	50.54	
Low	923	12.67	763	13.83	160	9.05	
Level of care							0.005**
Medical center	4,605	63.21	3,432	62.20	1,173	66.38	
Regional hospital	2,211	30.35	1,716	31.10	495	28.01	
Local hospital	469	6.44	370	6.71	99	5.60	
Department of hematology & oncology							0.019*
Without	4,066	55.06	3,118	56.51	948	50.78	
With	3,319	44.94	2,400	43.49	919	49.22	
Surgical operation							<0.001***
Without	5,943	81.58	4,559	82.62	1,384	78.32	
With	1,342	18.42	959	17.38	383	21.68	
Length of days	56.79±66.87		45.87±58.37		78.53±83.62		<0.001***
Medical cost (NT$)	387,795.92±512,441.07		323,169.80±453,916.71		589,610.82±620,984.80		<0.001***

P-value (category variable: Chi-square/Fisher exact test; continue variable: t-test). CCI_R = Charlson Comorbidity Index removed cancer.

*P<0.05

**P<0.01

***P<0.001

^#^ Catastrophic illness: Illness other than multiple myeloma contained the the Catastrophic Illnesses Patient Database.

When the two groups (survival vs. mortality) were compared based on the epidemiological characteristics, the results showed that the survival group using hemodialysis (dialysis) treatment ratio (54.95%) was significantly lower than the mortality group (81.44%) (P <0.001). In terms of gender, the proportion of women (40.99%) was significantly higher in the survival group than the mortality group (37.29%) (P = 0.003). In terms of age, the survival group was significantly younger than the mortality group (68.28 vs. 70.02 years; P <0.001). Moreover, the proportion of survivors aged ≤55 years was significantly higher than patients in the mortality group (15.69% vs. 12.39%; P <0.001) (Table 1).

The proportion of those experiencing catastrophic illness in the survival group was significantly lower than that in the mortality group (63.57% vs. 75.66%; P <0.001). In terms of admissions season, the proportions of survivors in the fall and winter were significantly higher than the mortality group (29.10% vs. 24.56% and 24.76% vs. 23.71%; P <0.001). Furthermore, the proportion of patients from northern Taiwan was significantly lower in the survival group than in the mortality group (46.67% vs. 58.63%; P <0.001), while that of patients from other regions was significantly higher in the survival group. In terms of the degree of urbanization, the proportion of patients from less-urbanized areas was significantly higher in the survival group than in the mortality group (13.83% vs. 9.05; P <0.001) (Table 1).

As for the hospital hierarchy, the rates for medical treatment at the regional hospital (inpatient) are much higher for survivors than for those in the mortality group (31.10% vs. 28.01%; P = 0.005). A significantly lower proportion of patients received hematological and oncological treatment in the survival group than in the mortality group (43.49% vs. 49.22%; P = 0.019). The proportion of patients undergoing disposal operation in the survival group was significantly lower than that in the mortality group (17.38% vs. 21.68%; P <0.001). The survival group had a significantly shorter hospital stay than the mortality group (45.87 days vs. 78.53 days; P <0.001). In terms of medical costs, the survival group had significantly lower expenditures than the mortality group (323,169 yuan vs. 589,610 yuan; P <0.001) (Table 1).

After controlling for other factors, the factors affecting hospital mortality in patients with MM include dialysis, gender, age, major injuries, season, urbanization, divisions, and the length of hospital stay.

The mortality risk of those in the inpatient dialysis group is 3.044 times that of those without dialysis (P <0.001); the male hospital mortality risk by adjusted is 1.162 times that of women (P = 0.012). Age is also a contributing factor, as patients over the age of 55 years have an in-hospital mortality risk by adjusted that is 1.511 times that of those ≤55 years of age (P <0.001). Hospital death due to catastrophic illness is 1.347 times the risk of a non-catastrophic illness (P <0.001), and the risk of in-hospital mortality was significantly lower in fall and winter than in spring (0.706 times and 0.827 times; P <0.001 and P = 0.023). The mortality risk was 1.443 times higher in urbanized areas than in less-urbanized areas and 1.726 times higher for in-hospital deaths than in the less-urbanized regions (P = 0.003; P <0.001). For in-hospital medical treatment with the Department of Hematology and Oncology, the mortality risk was 0.884-fold that of non–Hematology and Oncology (P = 0.049); the number of hospital days for each additional day in the hospital increased the mortality risk by 0.5% (P <0.001) (Table 1). Table 2 shows the effect of sex and age from 1997 to 2013, the number of hospitalizations, and in-hospital mortality ratio (percentage).

**Table 2:** Factors of multiple myeloma inpatient mortality by logistic regression in Generalized Estimating Equation (GEE) model.

	Adjusted OR (95%CI)	P
Hemodialysis		
Without	Reference	
With	3.044 (2.651–3.494)	<0.001***
Gender		
Male	1.162 (1.034–1.305)	0.012*
Female	Reference	
Age group (years)		
≦55	Reference	
>55	1.511 (1.272–1.796)	<0.001***
Low-income household		
Without	Reference	
With	1.613 (0.827–3.146)	0.161
Catastrophic illness^#^		
Without	Reference	
With	1.347 (1.171–1.549)	<0.001***
CCI_R	0.998 (0.942–1.057)	0.944
Inpatient season		
Spring (March-May)	Reference	
Summer (June-August)	0.898 (0.766–1.053)	0.187
Autumn (September-November)	0.706 (0.601–0.828)	<0.001***
Winter (December-Februrary)	0.827 (0.703–0.974)	0.023*
Urbanization level		
High	1.443 (1.134–1.811)	0.003**
Middle	1.726 (1.389–2.143)	<0.001***
Low	Reference	
Level of care		
Medical center	0.864 (0.655–1.140)	0.302
Regional hospital	0.897 (0.686–1.172)	0.426
Local hospital	Reference	
Department of hematology & oncology		
Without	Reference	
With	0.884 (0.782–0.999)	0.049*
Surgical operation		
Without	Reference	
With	1.050 (0.911–1.210)	0.497
Length of days	1.005 (1.005–1.006)	<0.001***

Adjusted OR = Adjusted Odds Ratio, adjusted for all variables in the table. CI = confidence interval. Nagelkerke R-square of model = 0.138. Outpatient location had multicollinearity with urbanization level. Medical cost had multicollinearity with length of d.

*P<0.05

**P<0.01

***P<0.001

^#^ Catastrophic illness: Illness other than multiple myeloma contained the the Catastrophic Illnesses Patient Database.

## Discussion

To the best of our knowledge, this is the first large-population based study regarding the epidemiology of MM in Chinese populations in recent years. Using the nationwide database, we included 7285 MM patients in Taiwan between 1997 and 2013. Huang has reported that the average incidence and the mortality per 100,000 people was 0.75 and 0.59 from 1979 to 2003 [8]. In our study from 1997 to 2013, the average incidence per 100,000 people was 1.83, which is compatible with recent reports that indicate an increase in incidence of MM in some Asian countries [8–9]. Further, the mortality was decreased, accounted for by an average of 0.44 per 100,000 deaths. This is due to the use of the novel agents, such as thalidomide, supported from 2009, or bortezomib from 2012 by the Bureau of National Health Insurance, which prolonged progression-free survival time and overall survival time. That is also a result of advanced supportive care in recent years. In our study, males and those >55 years of age were the most common of MM patients, and who may be predominantly older, and therefore related with more comorbidity. Kihyun Kim et al. has reported that males were more likely to have MM than females (55.6% vs. 44.4%), which is compatible with our study results [11]. Inpatients with MM were admitted more to the Hematology and Oncology ward, in the medical center and in the city. These adverse outcomes of renal failure and skeletal complications may be related to variations in the time required to obtain myeloma diagnoses. The median time between sign or symptom and myeloma diagnosis was 99 days [12–13]. Hence, MM was difficult to diagnose, especially in Asia and with hematologists/oncologists, who always worked in medical center in Taiwan, being required for diagnosis and management of MM. The proportion of a catastrophic illness was more in MM patients in our study. The previous study showed advanced stage of ISS stage was more in our hospital [14]. Delay diagnosis maybe the important factor of the high proportion of severely ill MM at diagnosis. However, this study was limited to prove the late diagnosis related high severely ill MM.

In our study, the ratios of the survival group using hemodialysis treatment and disposal operation were significantly lower than the mortality group. Hemodialysis treatment was 3.044 times the impact factor for hospital mortality in patients with MM. Many patients with myeloma develop renal dysfunction and skeletal complications, such as fracture or spinal cord compression, which decrease quality of life and increase mortality [13].

In terms of gender, age, and catastrophic illness for the survival group, patients who were female, aged ≤55 years and those with less catastrophic illness had significantly better prognosis in our study. The survival groups had significantly fewer hospitalization days and medical costs in our study. For those patients that were male and >55 years of age, catastrophic illness was a poor factor for hospital mortality in patients with MM.

The mortality group was significantly higher in the highly urbanized areas, in northern Taiwan, and in the medical center. It is widely known is that there is currently no cure for MM, thus, advances in therapy, such as autologous stem cell transplantation, radiation, and surgical care in certain cases, have helped to lessen the occurrence and severity of adverse effects of this disease and to manage associated complications [15–17]. The complicated management of MM was most performed in the medical centers, with many of the centers located in northern Taiwan and in the highly urbanized areas in Taiwan. The mortality numbers of patients from northern Taiwan was significantly higher than the other Taiwan. This is because the severe ill conditions of MM patient always transfer to medical center for management. Nine of 19 medical centers are located at the northern Taiwan. The mortality numbers of patients from low-urbanized area was significantly lower than the other middle- or high- urbanized area. This is the same reason that the severe ill conditions of MM patient always transfer to medical center for management. Nine of 19 medical centers are located at the northern Taiwan, which is high-urbanized area.

The MM patients for medical treatment at the regional hospital were 30.35%. However, the inpatient mortality rate of regional hospital was not different form that of medical center or local hospital (P = 0.302, 0.426, Table 2). Then the inpatient mortality rate of undergoing disposal operation was not different form that of non-operation (P = 0.497, Table 2).

This study had some limitations. First, because the NHIRD did not offer information regarding tumor stage and cause of death, we could not show stage-stratified survival rates and disease-specific survival rates. Second, we were unable to dealings patients directly to obtain additional information because of the anonymity ensured by the identification numbers. Third, we could not evaluate certain biological factors, such as genomics, because they were not encompassed in the NHIRD. Staging system of MM contains serum albumin, B2 macroglobulin and cytogenetic profiles, gene expression profiles, etc. However, this data was limited from the NHIRD. Finally, the results derivative from a cohort study are generally of a lower methodological quality than those from randomized trials because a cohort study design is subject to several biases related to adjustment for confounders.

In conclusion, those patients who were male and >55 years of age comprised those with the most common prevalence of MM in Taiwan. Hemodialysis treatment, male sex, old age, and catastrophic illness were independent predictors of poor factors for hospital mortality in patients with MM. To provide the most representative data on treatment results of MM patients achievable in Taiwan, we could prospectively collect nationwide data including detailed information. This could not only provide stage-stratified survival and disease-specific survival rates but also help to identify more clinical and biological prognostic indicators.
